# Cannabis arteritis

**DOI:** 10.11604/pamj.2017.26.53.11694

**Published:** 2017-02-01

**Authors:** Naoual El Omri, Rachid Eljaoudi, Fadwa Mekouar, Mohammed Jira, Youssef Sekkach, Taoufik Amezyane, Driss Ghafir

**Affiliations:** 1 Internal Medicine Department, Mohammed V Military Teaching Hospital, Rabat, Morocco; 2 Laboratory of Pharmacology and Toxicology, Faculty of Medicine and Pharmacy, Mohammed V University, Rabat, Morocco

**Keywords:** Cannabis, arteritis, digital necrosis

## Abstract

Cannabis is the most consumed psychoactive substance by young people. Chronic use of cannabis can lead to cannabis arteritis, which is a very rare peripheral vascular disease similar to Buerger's disease. It is affecting young adults, especially men, consuming cannabis. A 27-year old woman, with no particular past medical history except for long-term use of cannabis and tobacco developed a digital necrosis in the left hand. She denied using other illicit drugs. Doppler ultrasound examination of the upper limbs was unremarkable. Toxicological analysis revealed the presence of cannabis in both biological fluid and hair strand. Despite medical treatment, cessation of the cannabis and tobacco consumption and hyperbaric oxygen therapy, an amputation of necrotic parts was then required. This case shows the prolonged use of cannabis could be a risk factor for young adult arteritis. Faced with a rapidly progressive arteritis occurring in young adult, the physician should consider the history of use of cannabis. Hair analysis can be useful for confirmation of the chronic consumption of drugs.

## Introduction

Cannabis is the most widely used drug worldwide. It is made from the dried buds and flowers of the female Cannabis sativa plant. The major psychoactive component of marijuana is Δ9-tetrahydrocannabinol or Δ9-THC. Cannabis is smoked as hand-rolled cigarettes, pipes, cigars. It can also be mixed with other drugs such tobacco or cocaine [1]. Chronic abuse of cannabis may have many important health disorder such as psychiatric, respiratory and cardiovascular problems. Cannabis arteritis (CA) is a very rare peripheral vascular disease similar to Buerger's disease. It is presented as a peripheral necrosis most often of the lower limbs [2, 3]. Only about 50 confirmed cases between 1960 and 2008 were published in literature and most of them was men with CA in the lower limbs [4]. In this work, we report a case of a young women cannabis smoker with digital necrosis.

## Patient and observation

**Figure 1:**
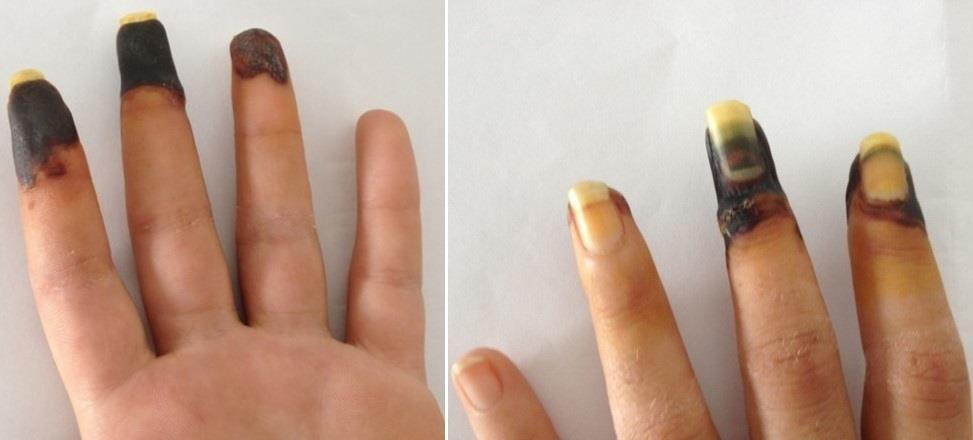
Digital necrosis

A 27-year old woman, Moroccan, consulted for a digital necrosis in the left hand (Figure 1). She is depressed but she has no family history of cardiovascular diseases including thrombosis. She had smoked approximately 20 cigarettes and five to ten cannabis hand-rolled cigarettes (joints) daily since the age of 15. She denied using other illicit drugs. Two weeks before, index, middle and ring finger of the left hand became bluish and extremely painful, and within days a necrotic lesion developed at the site of wounds. Clinical examination revealed a woman of healthy appearance and afebrile. Her weight was 48Kg, height 158 cm (BMI 22 Kg/m^2^) and blood pressure 110/60 mmHg. The rest of the examination findings are within normal limits except for dry necrosis of three fingers. Doppler ultrasound examination of the upper limbs and the supra-aortic trunks revealed normal arteries with normal blood flow. Magnetic resonance imaging and conventional angiography were not performed for our patient. Laboratory investigation revealed normal values for hematological parameters. Testing for thrombophilia including proteins S and C, antithrombin III, resistance to activated C protein, circulating anticoagulant antibody, anticardiolipid, b2 glycoprotein-1, homocysteinaemia were all in the normal range. Factor II and factor V Leiden mutation were normal. Cryoglobulins, polycythaemia and monoclonal gammopathy proved negative. Blood glucose, calcium and lipids were normal. Toxicological analysis using immunological methods revealed the presence of benzodiazepine, cannabis and cocaine. A wide screening of xenobiotics was made on whole blood and urine after liquid-liquid extraction were performed by liquid chromatography- tandem mass spectrometry (UPLC-TQD, Waters). This screening confirmed the presence of benzodiazepine (bromazepam), amisulpiride, bupropion, Δ9-THC and cocaine with its major metabolites: Benzoylecgonin and ecgonine-methyl-ester. To verify the chronic use of drug, a LCMSMS hair analysis for our patient was suggested. She agreed to the analysis and a strand of hair (12 cm) was cut from the vertex region of the scalp. For the segmental analysis, the hair sample was divided into two segments of 6 cm each. The hair analysis revealed the presence of cannabis metabolites in the two segments; the means of the concentrations were as follow: Δ9-THC (3.2 ng/mg), cannabinol (0.35ng/mg), cannabidiol (0.27 ng/mg). However, cocaine was not detected. Cannabis and tobacco consumption was immediately stopped. Treatment with anticoagulants, prostaglandin, acetylsalicylic acid and hyperbaric oxygen therapy was started. The treatment stopped the necrosis evolution, but no regression was observed. An amputation of necrotic parts was then required.

## Discussion

We report a case of young woman presenting a digital necrosis in the left hand, which seems to be strikingly related to the long term abuse of cannabis. Other causes of arterial diseases (e.g. occupational exposure to toxic agents, ergotism, thrombophilia, embolic heart disease, connective tissue diseases, inflammatory arteritis or ergotism) were excluded. Using the hair analysis, we also excluded other toxic arteriopathies encountered in drug addicts such as those due to cocaine, amphetamines or ergotamine, but we confirmed the chronic use of cannabis. Cocaine found in urine seem to be related to an occasional use. The use of hair analysis as a matrix for drug testing may help document chronic illicit drugs use. It is especially useful in situations when blood and urine specimens have not been collected on time. Cannabis use results in an altered mood. The psychoactive chemical compound is Δ9-THC, but more than 60 cannabinoids were characterized. Chronic cannabis use could also lead to arteritis. Cannabis arteritis was first reported by Sterne and Ducastaing in 1960 [5]. It is a very rare peripheral vascular disease similar to Buerger's disease and only a few cannabis users develop this complication, but the number is certainly underestimated. Both heavy and lighter users could develop the CA. This fact is probably due to the variable concentration of cannabis in the self-prepared products [6]. The pathogenesis of the CA is complex, Δ9-THC has a vasoconstrictor effect proved in animal studies. This effect may be mediated, in part, through a tyramine-like action on adrenergic nerve endings [7]. Arsenic may also be implicated in the pathogenesis of the CA because of tobacco co-intoxication. It seems to be a factor of vascular thrombosis and inflammatory arteritis. Arsenic could inhibit vascular endothelial growth factor and induce endothelial cell apoptosis. This theory would explain the decrease in prevalence of Buerger's disease with the refinement of tobacco. Finally a synergistic noxious effect of tobacco and cannabis seems likely [8]. Treatment of CA is for the patient to stop immediately and definitively cannabis and tobacco consumption. Anticogulant and vasodilatator drug (buflomedil, prostaglandins) can be given in the acute phase, followed by platelet aggregation inhibitor. Hyperbaric oxygen therapy can also be used [9]. Patient may have complete revascularization with treatment but advanced stages of necrosis may require amputation.

## Conclusion

Cannabis is the most widely illicit drug used in the world. If the acute toxicity of cannabis is low, chronic use can lead to serious complication such as CA. This condition is a serious peripheral vascular disease affecting young adults consuming cannabis. The risk of amputation is high if not treated. The diagnosis of CA should be suspected in of all young adults with peripheral necrosis.
